# Fractures of the Inferior Maxilla

**Published:** 1857-12

**Authors:** Frank Hastings Hamilton


// BUFFALO MEDICAL JOURNAL

AND

MONTHLY REVIEW.

VOL. 13. DECEMBER, 1857. NO. 7.

### ORIGINAL COMMUNICATIONS.

ART. I.—*Fractures of the Inferior Maxilla.* By FRANK HASTINGS HAMILTON, M. D.

*Division.* Of nineteen examples of fracture of this bone which have come under my observation, eighteen were broken through some portion of the body, namely, one perpendicularly through the symphysis; one through the symphysis and through the center of the body at the same time; one through the angle and center of the body; one through the body and ascending ramus; one through the angle and center of the body upon one side, and through the center of the body upon the opposite side; four through the angle only, and ten through the body only.

Of the whole number, eight were broken completely asunder at two or more points, constituting double and triple fractures; and of the remaining eleven, three were accompanied with detachments of portions of the alveola, and one with the detachment of a considerable fragment from the base.

From this analysis it will be seen that eleven of the nineteen, or more than one-half, were comminuted fractures. Seven were compound; not to include in this enumeration several examples in which the partial or complete dislodgment of a tooth, might entitle them to be called compound.

The two fractures through the symphysis were vertical, and eight of the remainder were known to be oblique. Malgaigne has remarked, also, that in fractures of the body of the bone the direction of the obliquity is generally such that the anterior fragment is made at the expense of the internal face of the bone, and the posterior fragment at the expense of the external face: this latter overriding the former. Buck, of New York, has seen the fragments in an opposite condition, requiring the use of the knife and the saw for their extrication.*

In thirteen examples of fractures through the body, not including fractures at the symphysis, the line of fracture has been observed to be eight times at or very near the mental foramen; twice between the first and second incisor; twice just behind the last molar, and once between the two last molars.

Symes, Liston, and Miller, have remarked, also, the greater frequency of fracture near this foramen, but Mr. Erichsen thinks he has seen it most frequently broken near the symphysis, between the lateral incisors, or between these teeth and the canine. Boyer observes that it is generally somewhat in front of the foramen; for which reason, as he thinks, the dental nerve is rarely torn.

Says Boyer, in his Traitê des Maladies Chirurgicals, "A fracture never takes place in the central point of the length of the jaw, called the symphysis of the chin; but when the solution of continuity occurs towards the middle of the bone, it is upon one or the other side of the symphysis, which remains always upon one of the fragments." An opinion which, however, he does not seem always to have entertained, since Richerand, in a report of his lectures has made him say that a fracture sometimes takes place "near the chin, but seldom so as to produce the division of the symphysis of that part, though it be not impossible." But many surgeons since his time, have noticed this fracture, and Malgaigne assures us that J. Cloquet has demonstrated its existence upon an anatomical specimen. In the two following cases the evidences were so complete that I do not myself entertain much doubt as to their character:

---

* New York Jour. Med., March, 1847. Proceedings of N. Y. Med. and Surg. Soc., Sept. 19, 1846.

An Irish laborer, aged 17 years, was thrown from a wagon, breaking the inferior maxilla on both sides through the body, and, also, exactly in the center, vertically, between the central incisors.

I dressed the jaw with a four-tailed bandage, but found great difficulty in bringing up the left fragment to a line with the right. I therefore closed the jaws; but finding that the left side still fell three lines below the right, I placed a pine wood wedge between the teeth on the right side, and drew the inferior maxilla up firmly. It now lacked only about half a line of being in place.

There did not appear to be, after this, much difficulty in maintaining quiet and apposition of the fragments; and I supposed, from repeated examinations, that they were in exact line, nntil four weeks after the fracture had occurred, when I discovered that the central fragments were lifted about two lines above the lateral, and, also, slightly carried back; and although union had not taken place, yet they could not be replaced by any moderate force. The bones united with this slight deformity. Four days later no motion was perceptible, and the displacement seemed to be rather less. From this time the dressings were discontinued.

A gentleman, aged twenty-five years, had his inferior maxilla broken by the kick of a horse. The left lateral incisor was completely displaced, and a large piece of the alveola detached.

Dr. S. G. Ellis, of Gowanda, N. Y., dressed the fracture, securing the loosened fragment by the main-spring of a watch, made fast to the teeth by a silver wire, and closing the mouth completely, without any interdental splint. Upon the outside he placed a pasteboard splint and bandages. On the fourth week a fragment exfoliated, and came out under the chin. The union was delayed some six or eight weeks.

I examined the jaw ten years after it was broken, and found the line of a vertical fracture exactly through the symphysis menti. The left half of the chin was slightly elevated, and the whole of that side of the shaft was smaller than the right. He could not close his teeth perfectly, yet he could close them sufficiently for the purposes of mastication.

Stephen Smith, of New York, has seen two examples,* Lonsdale mentions three,† and Gibson has seen one.‡

---

* New York Jour. Med., Jan., 1857, Hospital Reports.

† Practical Treatise on Fractures. By Edward F. Lonsdale. London: 1838, p 226.

‡ Institutes and Practice of Surg. By Wm. Gibson. Philadelphia: 1841, p. 261.

One ought not to be too confident, however, of the exact line of the fracture unless its existence can be demonstrated upon the naked bone, since a slight deviation to the one side or the other of the symphysis might not be easily detected in the living subject.

Velpeau, Fergusson, Gibson, Henry Smith and others, have remarked that a separation at the symphysis takes place usually in infancy or childhood. But in the seven examples whose ages I find reported, only one, a case mentioned dy Lonsdale, occurred in a person as young as ten years; in one, of the cases seen by myself the patient was seventeen years old, and the remainder have ranged from twenty-five years to sixty: and the average ages of all is thirty-two years.

I have seen one example of a fracture of the ramus, in a man twenty-three years old, who had been struck by a wooden block on the side of his face. The ramus was broken just above the angle, and the body was broken, also, obliquely near the symphysis. The intercepted fragment was carried inwards.* Ledran mentions the case of a child, ten or twelve years old, in whom the fracture was double also; one fracture having taken place through the body, and one extending obliquely from the root of the coronoid process to the neck of the condyle. The intercepted fragment was, however, so little displaced that the fracture of the ramus was not discovered until after death.† Malgaigne refers to this as the only example recorded; but Stephen Smith, of the Bellevue Hospital, has met with it four times, in one of which the ramus was broken on both sides; in two cases one ramus only was broken; and in one the body was broken on the right side and the ramus on the left.‡

In two of these examples the fragments were not displaced.

The coronoid process is so well protected by muscles and by the surrounding bony projections, that it is very rarely broken,

Houzelot mentions a case in which a fall from a height produced at the same time a fracture of both condyles, of both coronoid processes and of the symphysis.§

With this single exception, I am not able to find a recorded example of a fracture of this process.

At least eight cases have been reported of fracture of the condyles, in all

* Trans. Amer. Med. Assoc. Report on "Deformities after Fractnres," vol. viii., p. 385, case 17.

† Malgaigne, op. cit., p. 377, from Ledran, Observ. Chirurg, tom. 1, obs. viii.

‡ New York Jour. Med., Jan., 1857. Bellevue Hosp. Reports.

§ Malgaigne, op. cit., p. 400.

of which the separation occurred through the neck, viz., three by Ribes, two by Desault, one by Bérard, one by Houzelot, and one by Bichat. The fracture always occurring through the neck and just below the insertion of the external pterygoid muscle.

According to Malgaigne, the analysis of these cases shows two classes of examples: the one occasioned by falls or blows upon the chin, and producing a simple fracture of the neck of the condyle; the other, occasioned by injuries inflicted upon the side of the face, and producing a fracture of the neck on the side corresponding to that upon which the injuries are received, and at the same time a fracture of the body upon the opposide side. These two varieties seem to be about equally common.

In the case mentioned by Houzelot, and already cited, there existed at the same moment, a fracture of both condyles, of both coronoid processes and at the symphysis. Watson saw a man in the New York Hospital, who, three weeks before, had fallen from the yard-arm of a vessel, breaking his thigh and arm bones and also both condyles of the lower jaw. "His face was somewhat deformed by the retraction of the chin; the mouth could not be opened so as to protrude the tongue to any great extent beyond the teeth, and the teeth of the upper and lower jaw could not be brought into contact In attempting to move the jaw the patient experienced pain and crepitation just in front of the ears; the crepitation could easily be felt by placing the fingers over the fractured condyles. Nothing was done for the fractures of the jaw. In a few weeks the rubbing of the broken surfaces and attendant soreness ceased to trouble him; but the shape of the jaw and difficulty of opening the mouth, to any great extent, still remained unaltered."*

*Etiology.* The causes, in such cases as I have myself investigated, seem generally, if not always, to have been direct blows, in most instances inflicted by a club, or by the kick of a horse; in one instanee the blow was inflicted by the fist. In ten of eleven cases mentioned by Stephen Smith, the causes were direct blows. Examples of fracture of the inferior maxilla from indirect blows have, however, been mentioned by other surgeons, the angles of the bone being pressed together by the passage of a wheel, and the fracture taking place usually toward the symphysis.

We have already alluded to the observation of Malgaigne, that fractures of the condyles belong to two classes: the one being occasioned by falls upon the chin, and the other by blows upon the side of the face: the former acting as a counter force and the latter as a direct.

---

* New York Jour. of Med., Oct., 1840. Hospital Reports.

The coronoid process can only be broken by a direct blow.

*Symptoms.* Fractures of the body of the bone are characterized by the usual signs of fracture elsewhere, namely, displacement, mobility, crepitus, and pain.

The displacement is generally present; but its direction and amount vary according to the situation and course of the fracture, and also according to the violence and direction of the force producing the fracture.

I have never seen a case unaccompanied with some degree of displacement; Ledran and other surgeons, have seen such examples, where neither the periosteum nor mucous membrane have been torn.

Generally, in fractures of the body, the anterior fragment is depressed; and Malgaigne affirms that where an overlapping occurs, the anterior fragment lies, generally, within the posterior; a fact which he explains by the direction which the line of fracture usually takes, namely, from without, inwards and backwards, as we have already mentioned. In one instance, reported by me to the Amer. Med. Assoc., where the jaw was broken at the symphysis and also on both sides through the body, the central fragments were found, after about four weeks, lifted two lines above the lateral fragments, and also slightly carried backwards.* I have twice also met with examples in which the posterior fragments were inclined to fall inwards towards the mouth, a circumstance which seemed to indicate that the course of the obliquity was in a direction opposite to that which Malgaigne has observed to be most frequent. In each of these examples the jaw was broken upon both sides, by blows inflicted with a club, and the fractures were situated well back.† It is possible, however, that the position of the fragments was due rather to the direction and force of the impression than to the direction of the line of fracture.

As to the action of the muscles in the production of displacement, Boyer, S. Cooper, Erichsen and Malgaigne have observed that their action upon the anterior fragment is greater in proportion as the fracture is nearer the symphysis, and less in proportion as it approaches the angle. So that in the former case the attempt to close the mouth is sometimes attended with a depression of the anterior fragment, causing a separation of the fragments at their alveolar margins; while in the latter case, the attempt to close the mouth forcibly is occasionally attended with separation of the fragments along the line of the base.

---

* Trans. Amer. Med. Assoc., vol. viii., p. 380, 1855, case 6.

† Ibid, cases 1 and 10.

While I am not prepared to deny the accuracy of these observations, it is proper to notice that Liston finds the greatest displacement when the fracture is opposite the first molar, and I must confess that the fact as stated by Boyer and others, does not seem to admit of a satisfactory explanation; since the number, and consequently the power of those muscles which act upon the anterior fragment from below, is greatest at a point considerably remote from the symphysis. These muscles, namely, the digastricus, the genio-hyo-glossus, and the mylo-hoideus, with several other muscles which act less directly, all tend to depress the anterior fragment, and in some slight degree to carry it backwards, a direction which indeed it usually takes, and which it would probably always take if left alone to the action of the muscles. If the fracture has occurred through the angle, or at any point within the attachments of the masseter muscle, the action of those fibres which remain connected with the anterior fragment will sufficiently explain the fact that it is not now so easily depressed below the level of the posterior fragment: while the separation of the fragments along the line of the base when an attempt is made to close the jaw forcibly, is probably due to the loosening and partial dislodgment of some of the molars, which being pressed upwards act as a pivot upon which the fragments are made to bend.

Boyer affirms, also, that "the fractured portions are never deranged so as that one passes on the other, or in the direction of their length; for the action of none of the muscles of the lower jaw is parallel to the axis of that bone; besides, its extremities are retained in the glenoidal cavities of the temporal bones." But this theory is too exclusive, since the fragments may have become displaced in any direction independently of the muscular action. Moreover, the action of the muscles attached to the anterior fragment, although not parallel to the axis of the bone, does somewhat favor a displacement in this direction; and the action of the pterygoid muscles upon the posterior fragment still farther favors this form of displacement.

An overlapping of the fragments in the direction of the axis is, no doubt, exceptional, and in such examples as I have seen it, was very trivial. It occurred in the case marked III, in my report on Deformities after Fractures, the fracture being near the mental foramen; and in case II, the fracture being just anterior to the last molar; and also in case VI, where the bone had been broken through the center of the body on both sides and through the symphysis; but in neither case did the overlapping exceed two or three lines, and it was always easily overcome.

The mobility of the fragments is not so striking in these accidents as in fractures of the long bones, yet it is generally sufficiently marked, and especially where the bone is broken upon both sides at the same time. If only one side is broken, both motion and crepitus will be most easily detected by lateral pressure upon the posterior fragment, which being the smallest and the least supported by antagonizing muscles, will be found to be the most movable. If the fracture is upon both sides, mobility and crepitus will be most readily developed by seizing upon the anterior fragment and moving it gently up and down, while the finger rests upon the alveola within the mouth.

Sometimes a slight swelling or tenderness at some point of the alveola, or the loosening or complete dislodgment of a tooth, will indicate the point of fracture.

Pain, especially when the fragments are moved, is here more constant than in most other fractures, owing, perhaps, in part to the superficial position of the bone which renders the soft parts lying over it more liable to injury from the causes of fracture; but, also, in part to the lesions which the infra-maxillary nerve may have suffered. It is, indeed, a matter of surprise that injury to this nerve does not oftener seriously complicate these accidents, coursing, as it does, through so large a portion of the angle and body of the bone. One might naturally suppose that its complete disruption would often occasion paralysis of those portions of the face to which it is finally distributed, and that its partial lesions and contusions would create, in many cases, the most acute and constant suffering. It is rare, however, that we have present an amount of pain which might not be attributed to a severe shock, or a slight strain upon its fibres. I have myself never seen any extraordinary suffering distinctly attributable to an injury of the dental nerve after fracture, nor any degree of facial paralysis. Rossi relates a case in which convulsions followed this accident, and in which, as a final remedy, he proposed to expose and bisect the nerve; and Flajani saw a patient whose jaw had been broken, die in convulsions on the tenth day, the muscular contractions having commenced as early as the fourth day after the accident. The autopsy disclosed a rupture of the dental nerve, but no injury to the brain.

These two examples are, as far as I know, all which our records supply, in which grave results have been attributed to lesions of this nerve; and even here some doubt must remain whether the symptoms were not quite as much due to the immediate injury done to the brain as to the nerve.

Boyer explained the infrequency of severe injury to the dental nerve by the supposition that the "greater part of these fractures take place between the symphysis and the foramen by which this nerve comes out." An opinion which may be correct, but needs confirmation. I have seen the body or angle broken at points posterior to the mental foramen, and where the nerve lies within its bony canal, ten times, and in front of the mental foramen six times, and twelve times the point of fracture has not been stated with such accuracy as to enable me to say whether it was in front of or behind the foramen; of these latter, eight were said to be near the foramen. It may be fair to presume that at least one quarter of these eight were posterior, and this will leave just twelve as occurring where the nerve is within the canal, and an equal number at points without the canal.

I suspect that a better explanation may be found in the fact that the fragments seldom overlap, to any appreciable extent, and that even the displacement in the direction of the diameters of the bone is generally inconsiderable, or if it does exist it is easily and promptly replaced.

If the displacement is sufficient to occasion a complete disruption of the nerve, some degree of temporary paralysis in the portions of the face supplied by it must be inevitable; and, perhaps, this occurs oftener than it has been noticed, since during the confinement of the jaw by dressings, it is not likely to be observed, and after the lapse of a few weeks it will probably cease altogether.

Boyer remarks that when it is torn "the square and triangular muscles of the chin are paralyzed. The skin of that part and the internal membrane of the under lip preserve their sensibility, which it appears they owe to some threads of the portio dura of the seventh pair; but the paralysis of these muscles does not prove of itself that the jaw is fractured." Boyer has, however, noticed this result but once, and then in a case where the bone was broken upon both sides and the soft parts greatly contused. The triangular and square muscles were paralyzed, in consequence of which there was a slight contortion of the mouth. A. Bérard has also mentioned a case of vertical fracture occurring between the second and third molars, without displacement, which was accompanied with complete insensibility of the lip on this side throughout the space comprised between the commisure and the median line, and between the free border of the lip and the chin. The paralysis disappeared after a few days.*

To these signs now enumerated, we may add as occasional complications, rather than as diagnostic symptoms, salivation, swelling of the submaxillary and sublingual glands, abscesses, necrosis, &c. If the blow has been vertical upon the chin, and the direction of its force has been towards the articu-

* Malgaigne, from Gazette des Hôpitaux, 10 août 1841.

lations, the bony structure of the ear, and even the brain may have suffered serious lesions, which may be indicated by a deafness, or a roaring in the ears, by bleeding from the external meatus, and by fatal coma. Tessier saw a man who had received the kick of a horse exactly upon the center of the chin, breaking the bone on both sides, and who, in consequence, bled freely from his ears;* and Alix relates the case of a young man who, falling from a height and striking upon his chin, had broken his jaw. Insensibility immediately followed; convulsions also ensued upon the fourth day, and he died upon the sixth.†

If the fracture is at the symphysis, it is generally vertical, and either fragment may be found slightly displaced upwards or downwards. In one of the examples seen by myself, the left fragment fell three lines below the right, and in the other the right side had fallen about one line. In a case mentioned by Symes there was scarcely any displacement.‡ Liston remarks that it is usually slight. Erichsen and B. Cooper, have observed the same.

The signs which indicate a fracture through the angle have already been sufficiently considered when speaking of fractures of the body; from which they only differ in the less degree of displacement, and in the fact that the posterior fragments are a little more prone to fall inwards towards the mouth. I have noticed, also, that owing probably to the loosening and partial dislodgment of the last molar, it is sometimes difficult to close the mouth, the same as in fractures a little farther forward.

In the only example of fracture of the ascending ramus which I have seen, the bone being broken also through its body, the fracture of the ramus was easily recognized by both crepitus and mobility.

As to the signs which indicate a fracture of the coronoid process, I am only able to infer them from its anatomical relations. There must be some embarrassment in the motions of the jaw, occasioned by the detachment of a portion of the fibres of the temporal muscle; and it is probable that an examination by the finger, within the mouth, would readily detect mobility and displacement.

A fracture through the neck of the condyle is characterized by pain at the seat of fracture, especially recognized when an attempt is made to open or shut the mouth, by embarrassment in the motions of the jaw, by crepitus,

---

* Malgaigne, p. 383 and 386; from Jour de Méd., 1789, tom. 79, p. 246.

† Malgaigne, p. 386; from Alix, Observata Chir., fascic. 1, obs. 10.

‡ Amer. Jour. Med. Sci,, vol. xviii, p. 243.

which may usually be felt or heard by the patient himself, by mobility and displacement.

The upper fragment, if disengaged from the lower, is drawn forwards, upwards and inwards by the action of the pterygoideus externus; and it is felt not to accompany the movements of the lower fragment.

The lower fragment is at the same time drawn upwards, in consequence of which the lower part of the face is distorted: a circumstance first noticed by Ribes, and which supplies an important diagnostic mark between a fracture of one condyle and a dislocation. In dislocation the chin is commonly thrown to one side, but it is to the side opposite that on which the dislocation has occurred, while in fracture the chin is drawn to the same side.

*Prognosis.* Physick, of Philadelphia, saw a case of non-union of the body of this bone, which had existed nine months.* Dupuytren mentions a case which had existed three years.† Horeau has recorded one example in a man who had received a gunshot wound through his face;‡ and Stephen Smith, of New York, reports a case of fracture of both the body and ramus, in a man 45 years old. The severity of the injury with the supervention of delirium tremens, prevented the application of dressings until the thirteenth day. On the twentieth day about a pint of blood was lost by hæmorrhage from the seat of fracture. He remained in the hospital one hundred and thirty-seven days, and was finally discharged, the fragments not having yet united.§ Malgaigne says that Boyer has seen several examples, but I know of no other cases which have been recorded. In no instance under my observation, has the bone refused finally to unite, although I have seen the union delayed six, seven, ten, and even eleven weeks or more.‖ In three of these cases the fractures were either compound or comminuted; but in the case last mentioned, the fracture was simple, the delay in the union being due to a feeble condition of the system, and in part, perhaps, to neglect of proper treatment.

The infrequency of nonünion after this fracture, is a fact worthy of especial attention, because of the extreme difficulty, if not actual impossibility, in many cases, of preventing motion between the fragments, by any mode

---

* Phila. Med. & Surg. Jour., vol. v.

† Lecons orales.

‡ Malgaigne, from Jour. de Med., par Corvisart, etc., tom. x, p. 195.

§ Smith, New York Jour. of Med. & Surg., Jan., 1857.

‖ My Report on Defermities after Frac., Cases 2, 14, 15, 18.

of dressing yet devised. Any one who has observed attentively, must have seen, not only that his dressings are more often found disturbed and loosened, than in the case of almost any other fracture, unless it be the clavicle, and thus the fragments have been through all the treatment subjected to fiequent changes of position; but, also, that even while the dressings remain snugly in place, the patient seldom is able to perform the necessary acts of deglutition, or to speak, even, without inflicting some motion upon the fragments.

Indeed, the rapidity as well as certainty with which this bone unites, has, I think, been observed by other surgeons, and I have myself noticed one instance, in an adult person, in which the bone was immovable at the seat of fracture, on the seventeenth day, and, perhaps, earlier. In other instances, the union has been speedily effected after the removal of all dressings.

It would be very unsafe from these few cases, to deduce any conclusions as to the effect of slight motion in retarding the union of broken bones. Yet I shall take the liberty of referring to some remarks upon this subject, published by myself, in this Journal, vol. x, p. 142, in connection wi:h an article entitled "New Mode of Treating Ununited Fractures of the Humerus."

The amount of deformity resulting, also, from these fractures is usually very trifling, whatever treatment has been adopted. Seven of the nineteen examples seen by me, are recorded as resulting in some degree of imperfection, but one of these cases was complicated with other injuries of which the patient died in a few days, and one was a case of delayed union. Only five of the united fractures are imperfect, and in none of these is the imperfection such as to be noticed in a casual examination of the face. The deformity which is usually found, is a slight irregularity of the teeth, produced, in most cases, by a falling of the anterior fragment, and in one case by a slight elevation of the anterior fragment. But even this does not always interfere with mastication, and would often pass unnoticed by the patient himself. It is probable, too, that time, and the constant use of the lower jaw in mastication, will gradually effect a marked improvement in the ability to bring the opposing teeth into contact. I think I have observed this in several instances.

Chelius remarks that in "double or oblique fractures it is very difficult to keep the broken ends in their proper place; deformity and displacement of the natural position of the teeth commonly remains."

In the second example of fracture through the symphysis mentioned by me, the left fragment remained slightly elevated, and he could not close his teeth perfectly, yet he could close them sufficiently for the purposes of mastication. It is probable, however, that ordinarily no difficulty will be experienced in accomplishing a perfect cure when the separation has taken place only at the symphysis.

In fractures of the condyles more care is requisite to retain the fragments in apposition, and sometimes it may be found to be impossible. Richerand mentions the case of a man who having been three months in the Hospital De la Charité, for a double fracture of the lower jaw, one fracture being near the middle, and the other near the right condyle, left before the cure was complete. Seven or eight months after he called upon Boyer, who extracted from a fistula in the meatus auditorius externus, a bony mass, which had evidently the form of the condyle.* Bichat mentions a similar case as having come under the observation of Desault:† possibly it was the same which Boyer saw. Ribes says that a Parisian surgeon treated a double fracture of the jaw in a gentleman, one fracture being through the body and the other through the neck of the condyle; and in spite of the most assiduous and skillful attention, the patient recovered with a lateral distortion of the jaw, occasioned by the displacement of the fragments.‡ Ribes himself had to treat an accident of a similar character, and notwithstanding all his care, the result was the same as in the other example just cited.§

The proximity of this fracture to the articulating snrface may occasion contractions of thel igaments about the joint: and a degree of embarrassment to the motions of the jaw has followed in the experience of Desault and others, even when the cure has been most complete; but this has usually remained only for a short period.

Sanson asserts that when the coronoid process is broken, the fracture never unites; but that mastication is performed very well, the masseter and pterygoid muscles then fulfilling the office of the temporal.‖

---

* Boyer, Lectures on Dis. of Bones, p. 53, Phila. ed. 1805.

† Desault, Treatise on Fractures and Luxations, Phila. ed., 1805, p. 3.

‡ Malgaigne, op. cit., p. 402.

§ Ibid, p. 402.

‖ S. Cooper's First Lines, Amer. ed., 1844, vol. ii, p. 311

(To be Continued.)

